# Deep Learning and 5G and Beyond for Child Drowning Prevention in Swimming Pools

**DOI:** 10.3390/s22197684

**Published:** 2022-10-10

**Authors:** Juan Carlos Cepeda-Pacheco, Mari Carmen Domingo

**Affiliations:** Department of Network Engineering, BarcelonaTech (UPC) University, 08860 Castelldefels, Spain

**Keywords:** deep learning, 5G and beyond, child drowning prevention, network slicing architecture

## Abstract

Drowning is a major health issue worldwide. The World Health Organization’s global report on drowning states that the highest rates of drowning deaths occur among children aged 1–4 years, followed by children aged 5–9 years. Young children can drown silently in as little as 25 s, even in the shallow end or in a baby pool. The report also identifies that the main risk factor for children drowning is the lack of or inadequate supervision. Therefore, in this paper, we propose a novel 5G and beyond child drowning prevention system based on deep learning that detects and classifies distractions of inattentive parents or caregivers and alerts them to focus on active child supervision in swimming pools. In this proposal, we have generated our own dataset, which consists of images of parents/caregivers watching the children or being distracted. The proposed model can successfully perform a seven-class classification with very high accuracies (98%, 94%, and 90% for each model, respectively). ResNet-50, compared with the other models, performs better classifications for most classes.

## 1. Introduction

Drowning is a major health problem worldwide. According to the World Health Organization (WHO, Geneva, Switzerland), in 2015, around 360,000 people died from drowning [1]. More than half of these deaths are of people younger than 25.

The WHO Global report on drowning [2] states that the highest rates of drowning deaths occur among children aged 1–4, followed by children aged 5–9 years. In fact, in countries like Australia, drowning is the leading cause of unintentional injury death in children aged 1–3 years, and in the USA, drowning is responsible for more deaths among children aged 1–4 years than any other cause (except birth defects) [3]. Furthermore, drowning is the third leading cause of death worldwide for those aged from 5 to 14. In the Western Pacific Region, children aged 5–14 years die more frequently from drowning than from any other cause.

Drowning happens quickly and quietly and its signs often go unnoticed. Young children can drown silently in as little as 25 s, even in the shallow end or in a baby pool [4]. For all of these reasons, it is important for parents and caregivers to actively supervise their children around water, even if lifeguards are present.

The same report identifies the absence of or inadequate supervision as key risk factors for the drowning of children [1]. Another report [5] from the Royal Life Saving Society Australia (RSLA, Sydney, Australia) linked distracted parents to 77.8% of drownings in children aged 5–9 years in public and commercial pools between 1 July 2005 and 30 June 2015. In the cases of drowning without supervision, the parent or caregiver of the child was missing, or physically near the child but distracted (talking to another adult or attending to another child in his/her care). Furthermore, the German Lifeguard Association (DLRG, Bad Nenndorf, Germany) (the biggest organization of its kind in the world) reported that more than 300 people died in Germany during 2018 (from the beginning of the year through the summer) and associated the growing number of children drowning to their parents’ obsession with mobile phones [6]. In addition, Royal Life Saving Australia reported that, between 2002 and 2017, 447 children under the age of four drowned. Roughly 5% of those deaths were a direct result of a failure to supervise owing to the use of electronic devices (smartphone, tablet, laptop, and so on) [7].

In order to solve the problem of inadequate child supervision, in this paper, we propose a novel 5G and beyond child drowning prevention system based on deep learning that detects and classifies distractions of inattentive parents or caregivers. It can be deployed in indoor swimming pools or outdoor locations such as beaches or aquatic recreation locations aided by unmanned aerial vehicle (UAV) (drones). The system detects distracted parents/caregivers in charge of a minor and alerts them to concentrate on the supervision task. A 5G network slicing architecture for child drowning prevention has also been introduced. To the best of our knowledge, this is the first paper that aims to avoid child drowning by detecting and classifying distractions of parents in charge of a minor in aquatic recreational spaces; it is also the first paper to use digital technologies such as artificial intelligence and modern communication technologies (such as 5G and beyond) to detect and alert distracted parents or caregivers. The main contributions of this study are as follows:

- The proposal of a real-time distraction detection system that takes place in an aquatic recreational environment (swimming pools).
- The collection of our own distracted parent/caregiver image dataset by harvesting images of real people at a swimming pool being distracted or supervising children.
- The implementation and evaluation of three types of well-known convolutional neural networks (CNNs) for the classification and detection system to determine the most suitable architecture for distraction detection.
- The development of a voice alert system, pager, or wearable device that reminds the parent or caregiver to focus on the task of child supervision.

The experimental results prove the feasibility of the child drowning prevention system. The proposed model can successfully perform a seven-class classification with very high accuracies (98%, 94%, and 90% for each model, respectively).

The paper is structured as follows. In Section 2, we introduce our proposed 5G-enabled child drowning prevention system. In Section 3, we identify the most relevant key performance indicators (KPIs). In Section 4, we explain the 5G-service-based architecture. In Section 5, we discuss the proposed 5G network slicing architecture for child drowning prevention from a technical perspective. In Section 6, we briefly describe the convolutional neural network architectures used in this research. The experiments and results are presented in Section 7. Finally, Section 8 concludes the paper and highlights some future research directions.

### Related Work

Monitoring and supervision at swimming pools or aquatic recreation locations has drawn the attention of the research community [8], particularly for drowning prevention and early detection of possible drowning [9,10].

Some proposed drowning detection systems [11,12,13] employ underwater cameras to detect motionless drowned victims sunk at the bottom of the pool using techniques such as background extraction [13], which consists of detecting the moving objects by identifying the difference between the current frame and a reference frame, often called a ‘background image’ or ‘background model’; however, these systems are limited to the victims that have sunk to the bottom of the pool, thus wasting precious time, as they are unable to detect the victims prior to them drowning.

Other proposed methods consist of overhead cameras mounted around the pool (such as our proposed system) [14,15,16]; these systems consist of two main parts: a vision component that can detect and track swimmers and an event-inference (water crisis) module that analyzes swimmer observation sequences for possible drowning behavior signals. Several studies have been carried out regarding the detection of swimmers based on overhead cameras [17,18]. This task is still challenging owing to disturbances at the water’s surface (e.g., water exhibits random homogeneous blob movements, which could be easily misidentified as foreground objects) [19,20]. In addition, lightning and color variations over time due to ambient brightness even further complicate automated monitoring based on video surveillance. Several works apply background subtraction to solve the swimmer detection problem [13,19,20]. Currently, the development of wearable devices has become a very common practice. It has allowed researchers to develop sensor systems to monitor the physiological signals of high-performance swimming athletes [21,22], to detect pre-drowning symptoms and alert rescue staff [23], and to supervise children. Wearable sensor systems for infants can perceive external threats such as falls or drowning; the methods and techniques applied in wearable sensor systems are analyzed and discussed in [24]. In [20], a real-time detection method for constant monitoring of swimmers at an outdoor swimming pool is presented. A background subtraction scheme is introduced, where the background has been modeled as a composition of homogeneous region processes. Furthermore, to solve the foreground (swimmer) detection problem, a devised thresholding scheme has been proposed to attain a good trade-off between maximizing target detection while minimizing background noises. In addition, to enhance the visibility of the foreground (swimmer), a pre-processing filtering scheme able to classify each pixel of a current frame into different pixel types has been proposed; this way, appropriate filtering actions such as color compensation can be applied when necessary. In [19], a background subtraction scheme based on motion and intensity information has been developed to identify swimmers in each video frame. Image pixels are classified according to motion as random/stationary, ripple, and swimming. A motion map is developed through the computation of dense optical flow that characterizes the motion contents of image pixels over a short sequence of video frames rather than a single image. Intensity information has been modeled using a block-based mixture of Gaussians (MoG). However, these systems ([19,20]) only specify how to detect a swimmer; they do not specify how to detect if he/she is drowning.

Current improvements in computing power have enabled the use of deep learning algorithms for human detection and other computer-vision-related problems. Most state-of-the-art object detectors use deep learning algorithms to extract features from input images (or videos) and perform classification and localization, respectively [25]. In [26], a method to detect swimmers in low-quality video using two convolutional neural networks (YOLOv2 and Tiny-YOLO) has been proposed. Our proposed 5G and beyond child drowning prevention system is also based on deep learning (convolutional neural networks), but focuses on the detection of distracted parents/caregivers, not swimmer detection (as in [26]). In [27], a real-time vision system to detect drowning incidents using overhead cameras at an outdoor swimming pool is presented. The system uses a model comprising data fusion and hidden Markov modeling to learn of drowning events early. They focus on (1) foreground swimmer silhouette extraction and (2) behavioral recognition. The foreground detection module has already been reported in [20]. The system has analyzed water crisis episodes consisting of victims that suffer distress incidents (victims exhibit involuntary movements such as active struggling or waving [28]) and drowning incidents understood as suffocation. The detection of distress and early drowning episodes is based on visual indicators (instinctive response with repetitive arm movements of extending out and pressing down, perpendicular body (vertical up) in water with small movements in horizontal and diagonal directions). The experiments try to differentiate between three events (water crisis, treading, and normal swimming); the best testing errors obtained are 15.15% and 15.57%, with support vector machine (SVM) and reduced model (RM) classifier, respectively. Furthermore, the false alarm rate is at about one to five cases for each camera in a day. In addition, one challenge of their proposed system is that a drowning incident may happen in a way that is different from the learned instinctive drowning response model. In this case, it must be determined how the system will react to an event for which it is not trained [27]. Furthermore, specialists emphasize that drowning happens quickly and quietly, and its signs often go unnoticed (see Section 1). For this reason, in our current paper, we propose a novel technique to detect child drowning episodes that focuses on the caregivers of the children. To improve swimming pool safety, we use deep learning to detect a distracted caregiver of a child in a swimming pool, similarly to the detection of drivers’ distractions on the road. The behavior of a driver is essential for traffic safety. On-road distractions deteriorate the driver’s performance and may lead to the loss of vehicular control and traffic accidents. A distraction is anything that diverts the driver’s attention from the primary task of navigating the vehicle and responding to critical events. The authors in [29,30,31] use deep learning to detect distracted driver behaviors such as texting, operating the radio, drinking, fixing hair and makeup, talking on the phone, and so on.

## 2. The Proposed 5G and Beyond Child Drowning Prevention System

In the proposed scenario, families need to register when they arrive at the swimming pool. A facial image of each family member is acquired to recognize them. The swimming pool database registers the age of each child and links the photos of the children with their parents and/or other family member/s. The family decides who is going to be the primary caregiver that is going to watch the children and be responsible for their safety inside the swimming pool and a pager is given to him/her. This task can be shared between the parents (or other family members 18 years or older) simultaneously, which means that none of them should be distracted. It is also possible that there is only one primary caregiver during a certain time slot and another during the next time slot (e.g., the father is the primary caregiver from 15:00 to 17:00 and the mother from 17:00 to 19:00).

**Figure 1:**
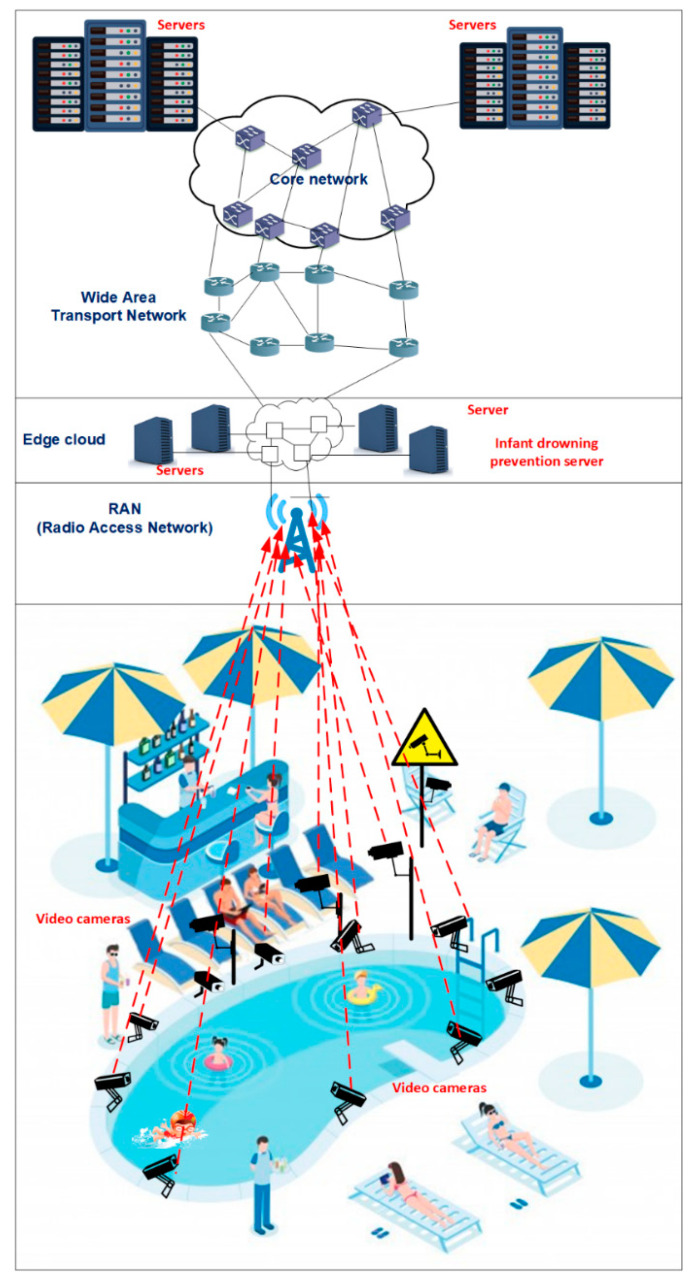
Proposed 5G-enabled child drowning prevention system.

After all of these decisions are made using the swimming pool app, the family can access the swimming pool area. The proposed 5G and beyond child drowning prevention system is shown in Figure 1.

If the primary caregiver decides to supervise the children outside of the pool, a specific seat will be assigned to him/her close to the swimming pool. This guarantees that he/she will have a good sight of the swimming pool to supervise the children. In addition, a video camera will be directly facing him/her to detect distractions. The cameras are strategically located at an optimal distance in a way not to obstruct people. In the case of multiple primary caregivers, the same or multiple video cameras can be facing them. Real-time video will be transmitted to the command center. Distractions of primary caregivers will be detected using a deep learning algorithm.

If the primary caregiver decides to supervise the child inside the pool, different video cameras mounted surrounding the pool will detect him/her using computer vision. For this purpose, a high-quality monitoring system is required that consists of video cameras with multiple high-end lenses that can zoom and steer around to detect critical details. The video cameras need to coordinate with each other to be able to track the primary caregivers at any time to detect possible distractions. The video cameras will identify the primary caregiver from different perspectives inside the pool. Automated analysis of the video footage will be carried out. A caregiver can be considered as ‘distracted’ if the convolutional neural network analyzes the images from all of the video cameras that are simultaneously capturing his/her behavior and he/she is characterized as being ‘distracted’ by most of them. That is, the images of the parents/caregivers are not combined, but the images from each camera are classified into a category. It is decided if the parent/caregiver is distracted or not by analyzing which category is repeated the most.

When a distraction event is detected, an alert will warn the primary caregiver so that he/she focuses on active child supervision. We assume that alerts will be sent immediately if the kids to supervise are 5 years old or under. For kids that can swim (usually older than 5 years), parents will be alerted if the convolutional neural network detects a continuous distracted behavior for more than 10 s, because drowning accidents happen very quickly. Alert messages can be sent to a pager. The pager lights up or vibrates in case the caregiver is distracted. Alert messages can also be heard through the swimming pool speakers located in the closest vicinity of the caregiver. Furthermore, lifeguards will also get these notification messages and act accordingly. This information will be, for example, useful if certain caregivers are notified several times; in this case, lifeguards can supervise the associated children much closer and talk to the parents/caregivers or take other necessary steps if no change in their attitude is observed.

## 3. Related Key Performance Indicators

The proposed 5G-enabled child drowning prevention system can be identified as a mission critical communications (MCC) service because it requires real-time and reliable communications for a large number of users, as well as strong security and pre-emption handling [32]. Table 1 summarizes the major key performance indicators (KPIs) for child drowning prevention. The end-to-end latency can be measured as the time interval required to send the packages from a source to a destination, measured at the application level.

Mission critical: A quality or characteristic of a communication activity, application, service, or device that requires low setup and transfer latency, high availability and reliability, the ability to handle large numbers of users and devices, strong security, and priority and pre-emption handling.

It would be possible for our use case to connect to the nearest edge server via Wi-Fi 7 (802.11be), because this standard will support a maximum throughput of at least 30 Gbps. Features operating at both the MAC (medium access control) layer and the physical layer (PHY) such as multi-access point coordinated beamforming, time-sensitive networking, and multi-link operation will bring Wi-Fi 7 latency performance into the sub-10 ms realm. These characteristics would be enough to support our high-throughput low-latency child drowning prevention use case. However, the IEEE task group announced draft 2.0 of 802.11be, and the final version will be released in 2024.

IEEE 802.11ax (Wi-Fi 6) received final approval from the IEEE Standards Board on 1 February 2021. This standard offers a theoretical speed of up to 9.6 Gbps and 10 ms latency. Wi-Fi 6 does not perform well in large-scale outdoor coverage scenarios and cannot meet the ultra-low latency requirements (<10 ms).

It has been shown in [33] that Wi-Fi 6 can achieve ultra-reliable low latency performance (i.e., <1 ms packet latency at 99.999% reliability) only when optimized and operating in a low load up to 0.16 bps/Hz that is not appropriate for our use case.

On the other hand, 5G can reach up to 10 Gbps (only slightly higher than Wi-Fi 6), but this technology has been designed to address the requirements of ultra reliable and low-latency communications (URLLC). URLLC has stringent requirements for capabilities such as latency, reliability, and availability. Some use cases include wireless control of industrial manufacturing or production processes, remote medical surgery, and transportation safety. It has been demonstrated in [33] that 5G NR (new radio)-FDD (frequency division duplex) has superior URLLC performance and meets the sub-ms delay requirement at >5× higher load than Wi-Fi 6.

Therefore, 5G is the appropriate technology for our use case thanks to its better latencies. The proposed system requires that real-time video is backhauled from the video cameras to the command center for remote control and analysis. The number of video cameras will vary depending on the size of the swimming pool. Moreover, 5G can be deployed in indoor swimming pools or even in outdoor locations such as beaches or aquatic recreation locations that extend several kilometers. In these cases where so many video images need to be processed as quickly and efficiently as possible, a 5G network is required to provide sufficiently high uplink data throughput and transmission reliability as well as sufficiently low latency. The short end-to-end latency will enable alert messages to be sent as fast as possible if necessary as drowning happens quickly. Reliability is critical to detect incidents, which means that performance should not be compromised irrespective of the channel conditions.

## 4. 5G Service-Based Architecture

**Figure 2:**
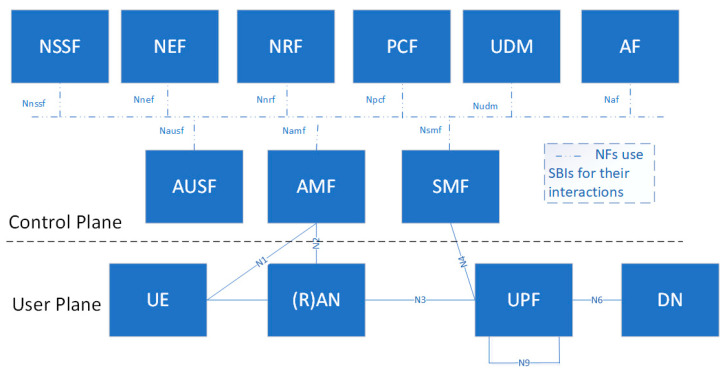
Service-based representation of the 5G non-roaming system architecture [34].

Next, the 5G system architecture of the non-roaming case is illustrated in Figure 2 [34]. The user plane (UP) and control plane (CP) are decoupled to obtain scalable and flexible deployments. Whereas the CP is used for network signaling, the UP carries only user traffic.

The user equipment (UE) in the user plane is connected to either the radio access network (RAN) or a non-3GPP access network (e.g., wireless local area network, WLAN) as well as to the access and mobility management function (AMF).

Next, we explain the network functions (NFs) of the 5G core network (see the upper part of the figure):

- Access and mobility management function (AMF): it is responsible for UE registration, reachability and mobility.
- Session management function (SMF): it offers UE IP address allocation and management, policy enforcement and quality of service, user plane function (UPF) selection, and control.
- User plane function (UPF): it is the anchor point for intra and inter radio access technology (RAT) mobility, packet routing, and forwarding.
- Policy control function (PCF): it integrates a policy framework for network slicing.
- Application function (AF): it is responsible for different services provided after the interaction with the core network.
- User data management (UDM): it is responsible for subscriptions and many services related to users.
- Authentication server function (AUSF): it performs the UE authentication service.
- Network slice selection function (NSSF): it offers an optimal selection of network instances serving the users.
- Network exposure function (NEF): it collects, stores, and exposes the services and capabilities provided by 3GPP NFs in a secure manner.
- NF repository function (NFR): it maintains and provides the deployed NF instances; it also supports the service discovery function.

## 5. A 5G Network Slicing Architecture for Child Drowning Prevention

Network slicing refers to the division of a physical network into multiple logical networks (network slices), so that each logical network can provide specific network characteristics for a particular use case. Network slicing provides services across multiple network segments and different administrative domains. A 5G slice can combine resources that belong to different infrastructure providers [35]. Network slicing is the best way for network operators to build and manage a network that meets the requirements from a wide range of users. Network slicing provides service flexibility and the ability to deliver services faster with high security, isolation, and according to the quality of service (QoS) requirements of the different applications. This way, network operators can manage their network resources efficiently and provide differentiated and scalable services.

Slices are isolated from each other, which means that faults or errors in one slice do not affect the proper functioning of another slice.

**Figure 3:**
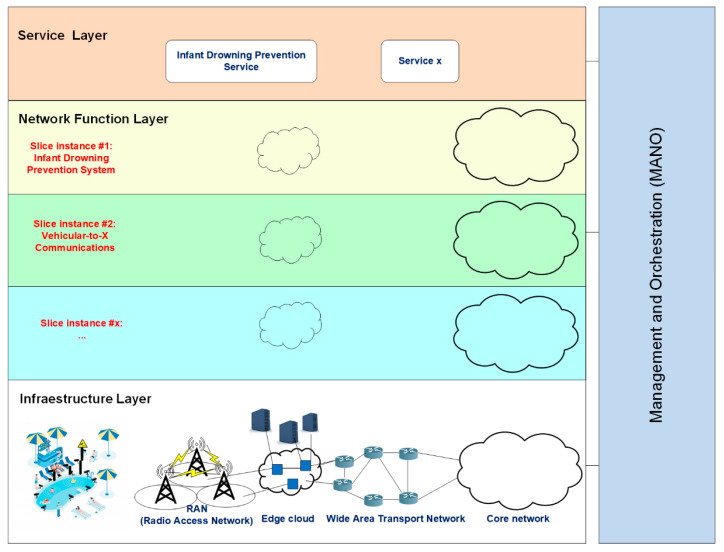
Network slicing architecture for child drowning prevention.

Next, we introduce the main design elements of our proposed 5G network slicing architecture for child drowning prevention (see Figure 3).

It is divided into three layers plus an additional management and orchestration layer, whose basic functionalities are summarized as follows:

Infrastructure layer: It refers to all of the parts of the physical network, because slices should be end-to-end. This layer includes the IoT networks, telecommunication networks, satellites, edge computing technologies, and the cloud. It provides the allocation of virtual or physical resources such as computing, storage, network, or radio.

We assume that all network devices are software defined networking (SDN)-enabled switches managed by SDN controllers that are able to program their routing tables.

The 5G core is generally divided into ‘core—user plane’ in charge of bearer delivery and ‘core—control plane (CP)’ in charge of control functions. Core—control plane will stay in the central cloud (network function virtualization, (NFV)), but ‘core—user plane (UP)’ will be distributed to its tens of edge nodes nationwide and be installed in edge clouds (NFV). Security, reliability, and latency will be critical for a 5G slice supporting the child drowning prevention case. For such a slice, all of the necessary (and potentially dedicated) network functions should be instantiated at the edge node. We consider that all the 5G core functions/units (UP) should be in the edge cloud close to the users. Multi-access edge computing (MEC) drastically reduces the latency between network nodes and remote servers in the cloud [36] because video processing servers are placed right where the core functions/units are located. This way, we can minimize the transmission delay to match the requirements of our delay-critical slice for such an MCC application. Furthermore, machine learning is crucial in supporting MCC by enabling a local decision making process at the edge servers [37].

Network function layer: It encapsulates all of the operations related to the configuration and life cycle management of the network functions that offer an end-to-end service. Network function virtualization (NFV) [38] and SDN (software-defined networking) [39] are two fundamental technologies to configure the virtual network resources. NFV decouples specific network functions from dedicated and expensive hardware platforms. This technology can provide software building blocks named VNFs (virtualized network functions) for the data plane that can be connected and chained according to the service type. SDN technology enables the separation of the control plane from the data plane to offer a flexible resource management.

Service layer: This layer provides a unified vision of the service requirements. Each service is represented by a service instance, which embeds all of the network characteristics that satisfy the SLA (service level agreement) requirements such as throughput or latency. A network slice instance (NSI) is a managed entity created by an operator’s network with a lifecycle independent of the lifecycle of the service instance(s) [40]. An NSI provides the network characteristics required by a service instance. It is also possible that an NSI is shared across multiple service instances of a network operator.

Based on the main KPIs (see Section 3) and functional requirements of our use case, child drowning prevention, we propose that the drowning prevention slice has ultra-reliable and low-latency communications (URLLC) requirements. URLLC use cases (such as mission-critical applications) have stringent latency, reliability, and availability requirements.

Management and Orchestration (MANO): It is the framework for the management and orchestration of all network resources (computing, networking, storage, and virtual machine) in the cloud. It comprises three functional blocks: NFV orchestrator (NFVO), VNF manager (VNFM), and virtualized infrastructure manager (VIM). NFVO performs on-boarding of new network service and VNF packages, network service lifecycle management, and resource management. VNFM manages the lifecycle of VNF instances. VIM controls and manages the lifecycle of virtual resources as requested by the NFVO in an NFV infrastructure (NFVI) domain.

## 6. Convolutional Network Models

Convolutional neural networks (CNNs): They were created out of the need to be able to process images effectively and efficiently; nowadays, they are also used for speech recognition. However, their strength is in image processing. Next, we describe the CNNs used in our research.

**Figure 4:**
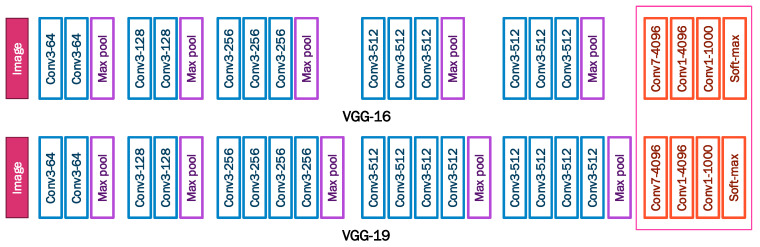
VGG-16 and VGG-19 architecture.

VGG model: This architecture was proposed by Karen Simonyan and Andrew Zisserman [41]; it was the winner of the ImageNet Large-Scale Visual Recognition Challenge 2012 (ILSVRC12). It was designed with 16 hidden layers in VGG-16 and 19 hidden layers in VGG-19 versions. The architecture processes input images of size 224 × 224 pixels with three channels for color images (RGB). The image is passed through five convolutional blocks (Figure 4). In VGG-19, the first two blocks incorporate two convolutional layers and the remainders incoporate four convolutional layers. Each convolutional layer uses 3 × 3 filters and rectified linear unit (ReLU) as an activation function; the convolutional blocks also incorporate maxpooling layers to reduce image size and prevent overfitting problems; the upper layers are composed of two full-connected layers with 4096 neurons each, at the top, one output layer for image classification into 1000 different categories.

**Figure 5:**
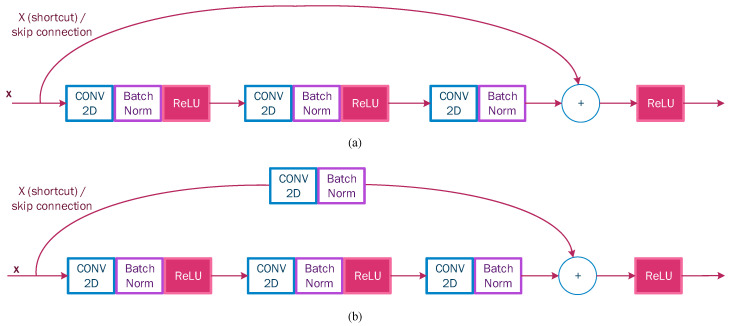
(**a**) ResNet identity block and (**b**) ResNet convolutional block.

**Figure 6:**
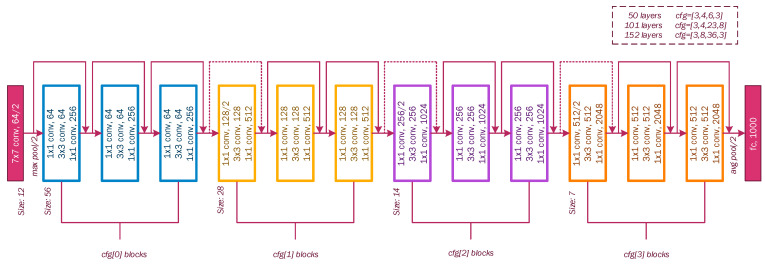
Configuration of residual network architecture, including ResNet-50, ResNet-101, and ResNet-152.

ResNet model: It is a type of advanced convolutional neural network; this model was proposed by Kaiming He in his 2016 document [42]. The ResNet-50 version consists of 50 layers. This model is based on the idea of residual and identity blocks that use skip connections (shortcut) (Figure 5), where the input is passed to a deeper layer. In other words, the simple deep convolutional neural network is inspired by VGG with 3 × 3 filters and a ReLU activation function, which is modified to become a residual network by adding skip connections to define residual blocks. On the top, the architecture contains a fully connected output layer with a softmax activation function for classification. Figure 6 shows the general configuration of the residual network architecture, including ResNet-50, ResNet-101, and ResNet-152.

**Figure 7:**
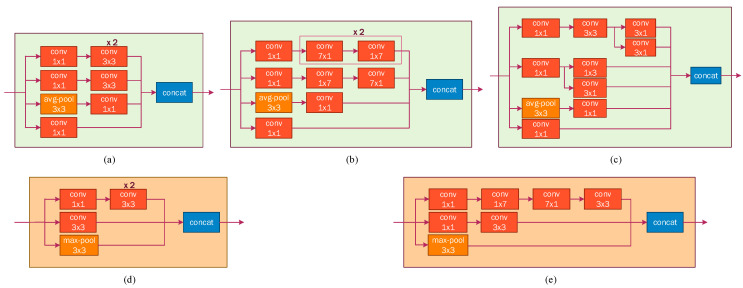
(**a**) Inception-A block, (**b**) inception-B block, (**c**) inception-C block, (**d**) reduction-A block, and (**e**) reduction-B.

Inception-v3 model: This convolutional neural network was developed by Google. The first version of inception, called “GoogLeNet”, was presented in the ImageNet Large-Scale Visual Recognition Challenge 2014 (ILSVRC14) [43]. This first version of the architecture is made up of 22 layers including convolutional, pooling, and a characteristic layer called inception; the latter is a type of convolutional layer, but it is characterized using only 1 × 1, 3 × 3, and 5 × 5 filters simultaneously (Inception blocks) (Figure 7); this way, the number of parameters to calculate is greatly reduced. This was achieved with what Google called bottlenecks, which were convolutional layers with 1 × 1 filters to reduce the complexity of the network. Google also includes auxiliary classifiers, intending to facilitate the propagation of the gradients backward and to reduce the cost involved Therefore, reducing the number of parameters and complexity resulted in a more powerful network.

**Figure 8:**
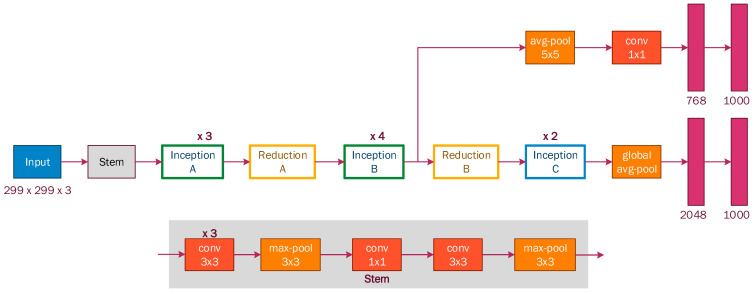
Inception-v3 architecture.

Figure 8 shows the inception and reduction blocks that were set for the third version of this architecture.

## 7. Experiments and Results

### 7.1. Dataset

The dataset is a collection of 38,000 images generated by us in the summer of 2019. The location of the video recording was the facilities of the Fontsanta swimming pool, located at Carrer del Marquès de Monistrol, 30, 08970 in Sant Joan Despí, Barcelona—Spain. Five primary caregivers (people in charge of the children) were involved in the development of these experiments. They were recorded on video, doing different activities (one video for each action related to each of the different categories) both inside and outside the water. The images captured from each video correspond to a specific category so that the images have been identified and labelled manually for each category. The capture was made taking into account that only the participants appear in the video to protect the privacy and confidentiality of other people who are at the swimming pool. The videos were recorded with high-resolution smart mobile devices (1920 × 1880), although the images are preprocessed according to the input data requirements of each model (224 × 224). The images were finally collected and classified into seven (7) categories:

- *I_distracted: * In the water distracted.
- *I_watching:* In the water watching the children.
- *O_distracted: * Out of the water distracted.
- *O_talk_cell:* Out of the water talking on a cell phone.
- *O_reading:* Out of the water reading a book.
- *O_chatting:* Out of the water chatting on a cell phone.
- *O_watching: * Out of the water watching the children.

To achieve a great performance during the training process with our own dataset, the videos were not shot from a single angle. Instead, they were shot from different angles, covering all potential perspectives of a caregiver. Furthermore, because the swimming pool is located outdoors, the varying lighting conditions throughout the day provide a richer dataset.

### 7.2. Experimental Settings

The dataset consists of approximately 38,000 images; it was split into two parts, keeping a ratio of 8:2, i.e., around 30,000 images for training and 8000 for testing. In addition, data augmentation was used to expand the training set and obtain better generalization. Data augmentation is a technique that expands our original training dataset virtually, through a random series of transformations from the original image, resulting in new plausible-looking images, in order to obtain a larger number of images for training. In computer vision, this technique became a standard for regularization, as well as to improve accuracy, generalization, and control of overfitting in CNNs. For this research, the techniques chosen are as follows: rescale = 1./255, rotation_range = 2, shear_range = 0.2, zoom_range = 0.2, and horizontal_flip = True.

**Figure 9:**
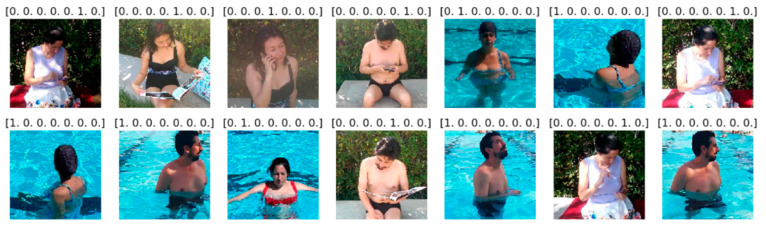
Image set of each category with their respective training labels.

**Figure 10:**
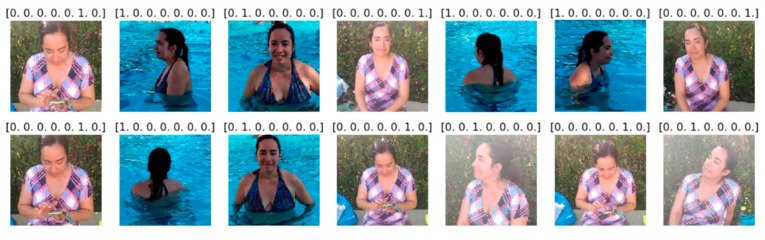
Image set of each category with their respective testing labels.

We have selected the images from a different subject for testing purposes in order not to contaminate the testing set. Figure 9 and Figure 10 show a set of images of each category with their training and testing labels.

The algorithms were implemented in several Jupyter Notebooks in version 6.0.3 installed with anaconda programs suite, developed in Python. The experiments were carried out on a Lenovo computer 2.9 GHz Intel (R) Xeon (R) processor with 72 GB RAM, without GPU.

We implemented three different algorithms using the preset models from the python Keras library; each one was specifically adapted to obtain optimal results after each training. The transfer learning technique was used (further details will be provided in Section 7.4) to take advantage of the pre-trained weights. Early stopping and dropout were implemented as techniques to avoid overfitting to achieve an improvement of the generalization capacity. Accuracy was selected during the training process as a metric to evaluate the performance of each algorithm.

The setup of each model to be used is detailed below.

### 7.3. Convolutional Neural Network Architectures

In this paper, experiments were performed to evaluate the proposed approach with three different CNN architectures: VGG-19, ResNet-50, and Inception-v3. Table 2 presents a summary of the configuration for each model. For all experiments, we used an image size of 224 × 224 × 3 and a batch size of 64.

#### 7.3.1. VGG-19

We implemented the VGG-19 version because it has a greater number of layers (deeper network) compared with the VGG-16 version mentioned above. It is made up of a 224 × 224 × 3 input layer, five convolutional blocks with kernel 3 × 3, ReLU activation function, without padding, and a maxpooling layer after each block followed by a flattened layer and two additional blocks; each additional block consists of a fully connected dense layer with 4092 neurons, a BatchNormalization layer, and a dropout layer. The last layer is a dense layer with a softmax activation function that contains seven neurons to classify our categories.

#### 7.3.2. ResNet-50

This model contains an input layer of 224 × 224 × 3, fifty convolutional blocks with their respective skip connections, followed by a global average pooling layer. At the top of the model, we have added two additional blocks; each block consists of a fully connected dense layer with 2048 neurons, a BatchNormalization layer, and a dropout layer. The last layer is a dense layer with a softmax activation function that contains seven neurons for our classification.

#### 7.3.3. Inception-v3

This model is composed of a 224 × 224 × 3 input layer, two convolutional blocks of three and two layers, followed by a maxpooling layer after each block. In the central part, it consists of several types of inception and reduction blocks, along with a global_average-pooling layer. At the top of the model, we added two additional blocks; each block consists of a dense layer fully connected with 2048 neurons, a BatchNormalization layer, and a dropout layer. The last layer is a dense layer with a softmax activation function that contains seven neurons for our classification.

### 7.4. Training

**Figure 11:**
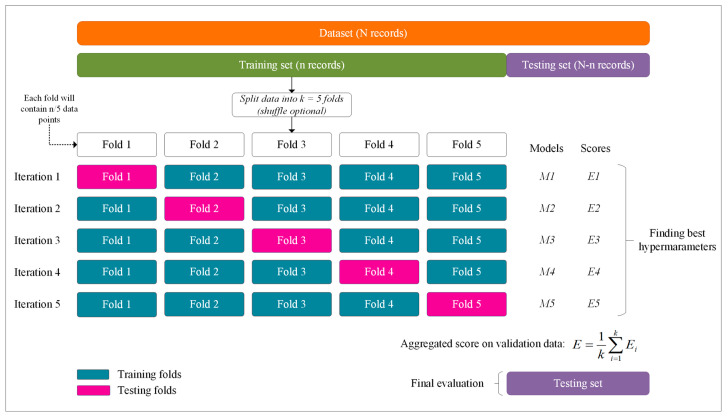
Use of each fold in the cross-validation process (fivefold representation).

The dataset consists of approximately 38,000 images (N records); it was split into two parts, keeping a ratio of 8:2, i.e., around 30,000 images for the training set (n records) and 8000 for the testing set (N−n records). For the training, we applied cross-validation. Cross-validation is a technique commonly used to validate machine learning models and estimate the performance of the model trained on unseen data. The most robust and widely used method of cross-validation is K iterations or K-fold cross-validation. This method consists of splitting the training dataset into K subsets (see Figure 11). During iterations, each of the subsets are used as validation data or testing folds and the rest (K−1) as training data or training folds. The cross-validation process is performed repeatedly for K iterations, with each of the subsets of validation data. The arithmetic average of the results of each iteration is finally performed to obtain a single result. This method is highly efficient as we evaluate it from K combinations of training and validation data, but it still has a disadvantage, that is, computationally, it is slow. However, the choice of the number of iterations depends on how large the dataset is. Cross-validation is most commonly used with K values ranging from 5 to 10. If the model (estimator) is a classifier and the target variable (y) is binary or multiclass (as in this research), the StratifiedKfold technique is used by default. This approach introduces stratified folds, i.e., by keeping the proportion of samples from each class in all folds. Therefore, the data from the training and testing folds are distributed equally. It is useful when unbalanced datasets are used. To evaluate the results, we used several metrics that are very common in machine learning applications for classification problems.

#### 7.4.1. Loss or Cost Function

A loss function is employed to optimize a machine learning algorithm. Several different cost functions can be used. Each of them penalizes errors differently. The loss function most commonly used in deep neural networks for classification problems is cross-entropy. In this research, we employed categorical cross-entropy. Categorical cross-entropy is a loss function that is used in multi-class classification tasks, where a sample can be considered to belong only to a specific category with a probability of 1 and to other categories with a probability of 0, and the model must decide which category each one belongs to.

#### 7.4.2. Transfer Learning and Early Stopping

A model can be trained from scratch when it is not very large or when the necessary computational capacity for its execution is available. On the other hand, it is possible to take advantage of the benefits of pre-established models and use them in new models. This technique is known as transfer learning; this means that it allows us to transfer learning from a pre-trained model such as VGG-19, ResNet-50, Inception-v3, and so on (pre-trained models for 1000 objects’ classification) and apply it to new classification algorithms. Furthermore, it is possible to unfreeze some pre-trained layers by adapting the model (fine-tuning) to re-train them along with the new fully connected layers; this method implies increasing the training time to avoid overfitting problems and to obtain optimal performance from the algorithm.

A popular technique to overcome overfitting is early stopping. For this purpose, at each iteration, the training set is divided into training and validation folds. The training folds are used to train the model and the validation folds are used as validation data at each iteration. In each training of the model, the validation folds help us to verify the accuracy of the model at the end of each epoch. Therefore, as soon as the test error starts to increase, the training is stopped.

### 7.5. Evaluation Metrics

To evaluate the results, we used several metrics that are very common in machine learning applications for classification problems.

#### 7.5.1. Accuracy

It is defined as the number of predictions made correctly by the model of the total number of records.

(1) $$accuracy=\frac{TP+TN}{TP+FP+FN+TN}$$

where *TP* represents true positives, *TN* represents true negatives, *FP* represents false positives, and *FN* represents false negatives.

#### 7.5.2. Precision

We evaluate our data for its performance of “positive” predictions.

(2) $$precision=\frac{TP}{TP+FP}$$

#### 7.5.3. Recall (Sensitivity) (True Positive Rate)

It is calculated as the number of correct positive predictions divided by the total number of positives.

(3) $$recall=\frac{TP}{TP+FN}$$

#### 7.5.4. Specificity (True Negative Rate)

It is calculated as the number of correct negative predictions divided by the total number of negatives.

(4) $$specificity=\frac{TN}{TN+FP}$$

#### 7.5.5. F1 Score

It is the weighted average of precision and sensitivity. Therefore, this score takes into account both false positives and false negatives.

(5) $$F1\;score=2\times \frac{\left(precision\times recall\right)}{\left(precision+recall\right)}$$

#### 7.5.6. Loss

Loss is the value that reflects the sum of errors in our model. It indicates whether the model is performing well (high value) or not (low value); on the other hand, the accuracy can be defined as the number of correct predictions divided by the number of total predictions.

Therefore, if we analyze these two metrics together (loss and accuracy) (see Table 3), we can deduce more information about the model performance. If loss and accuracy are low, it implies that the model makes small errors in most of the data. However, if both are high, it makes large errors in some of the data. Low accuracy but high loss would mean that the model makes large errors in most of the data. However, if the accuracy is high and the loss is low, then the model makes small errors in only some of the data, which would be the ideal case.

### 7.6. Experimental Results

After training with different configurations in the upper layers of each model, the following results were obtained.

#### 7.6.1. Loss and Accuracy

For training, cross validation was performed; therefore, the early stopping technique was used to avoid overfitting (as mentioned above); thus, training is stopped once it has reached the maximum accuracy value. Furthermore, the checkpoint was used to save the weights of the trained model when a new maximum value arises and we can load it in the future. Table 4 shows a summary of the accuracy and loss for the training and testing of each model. We can see that, for training, all models achieve an accuracy above 99% and ResNet-50 achieves a higher loss value compared with the other two models. Furthermore, for testing, ResNet-50 achieves the highest accuracy, but also the largest loss of 98% and 0.3203, respectively. VGG-19 achieves an accuracy of 94% and the lowest loss of 0.0039 and, finally, Inception-v3 achieves an accuracy of 90% and a loss of 0.0364. Based on the accuracy, ResNet-50 has developed much better performance compared with the other trained models.

Table 5 shows the accuracy achieved by each model with each of the classification categories (seven), evidencing the performance in more detail. VGG-19 achieves an accuracy of 100% for I_watching and O_reading categories, an average accuracy of 97.42% for the remaining categories, and a lower value of 72.73% for the O_chatting category. Similarly, ResNet-50 achieves an accuracy of 100% for the I_watching and O_talk_cell categories and the worst result for the O_distracted category, with an accuracy of 95.4%. On the other hand, Inception-v3 achieves a high accuracy of 98.68% for the I_distracted category and a lower accuracy of 66.6% for the O_talk_cell category.

As this research work focuses on parental distraction detection for child drowning prevention, the “In the water watching the children” (I_watching) and “Out of the water watching the children” (O_watching) categories are the most relevant ones to detect if parents/caregivers are really supervising their children. All of the other categories just represent that the caregivers are distracted and should be warned. For I_watching, the VGG-19 and ResNet-50 models achieve an accuracy of 100% and Inception-v3 achieves an accuracy of 96.83%. Likewise, for O_watching, the VGG-19 and ResNet-50 models achieve an accuracy of 99.61% and Inception-v3 achieves an accuracy of 84.42% (Table 4).

#### 7.6.2. Precision, Recall, and F1-Score

Accuracy should not be considered as a single metric for measuring model performance when using an unbalanced data set, as it counts the number of correct predictions regardless of the type of category, leaning towards the majority categories. In other words, from a dataset of 100 cases where 95 belong to the category “a” and five to category “b”, if only all the cases in the first category are correctly predicted, an accuracy of 95% would be obtained. This value is misleading because 95% refers only to the correctly predicted values of one category (50% of the total predictions).

Because our data are unbalanced, we also consider other metrics such as recall, precision, specificity, and F1-score to evaluate our results. Table 6 shows the values obtained in every category based on the above-mentioned metrics for VGG-19. F1-score is the harmonic mean of precision and recall and it takes into account both false positives and false negatives. The VGG-19 model performs well because it achieves an accuracy between 96% and 99% for most categories and a smaller accuracy of 84% for the *O_reading* category. We can also observe that, for the most relevant categories (I_watching and O_watching), this model reaches an F1-score of 98%, demonstrating good performance in training.

Table 7 shows a summary of the already mentioned metrics in every category for the ResNet-50 model. It achieves an F1-score between 97% and 99% for all categories. It should be pointed out that this model reaches an F1-score of 98% and 99% for the most relevant categories (I_watching and O_watching), which is the best performance of the three models.

Finally, Table 8 shows a summary of the already mentioned metrics in every category for the Inception-v3 model. This model achieves an F1-score between 91% and 98% for most categories, and a minimum F1-score of 79% for the O_talk_cell category. In this case, the Inception-v3 model achieves an F1-score of 98% for the I_watching category, but the lowest F1-score of 84% for the O_watching category (most relevant categories).

According to this, we conclude that the ResNet-50 model shows excellent performance for this classification problem, reaching F1-scores of 98% and 99% in the I_watching and O_watching categories, respectively (see Table 7). However, the VGG-19 model with a value of 98% in the mentioned categories shows a solid performance as well (see Table 6).

#### 7.6.3. Confusion Matrix, False Positive Rate, and False Negative Rate

**Figure 12:**
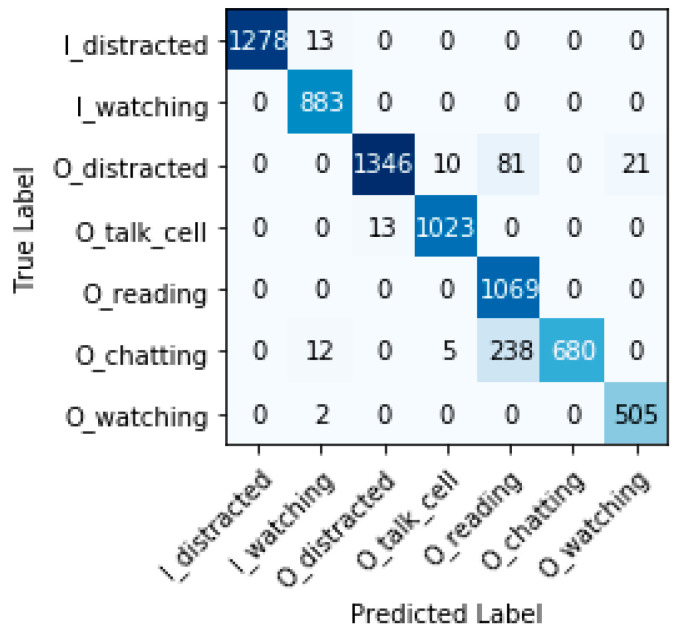
Confusion matrix VGG-19.

**Figure 13:**
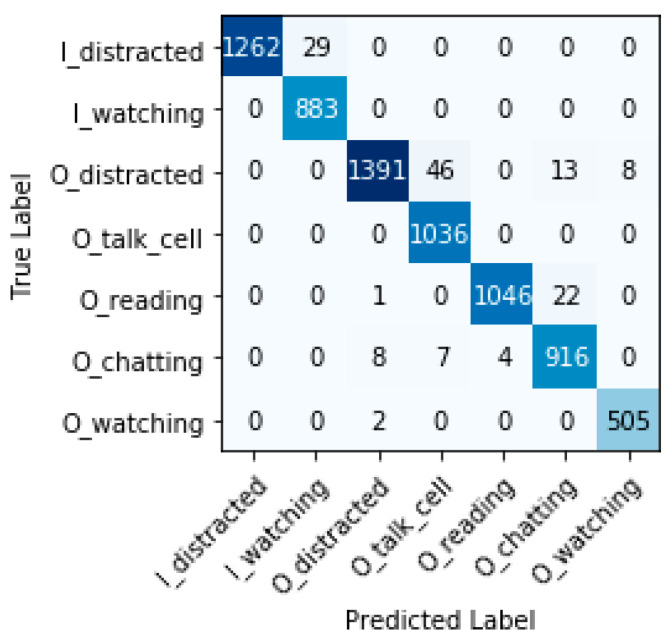
Confusion matrix ResNet-50.

**Figure 14:**
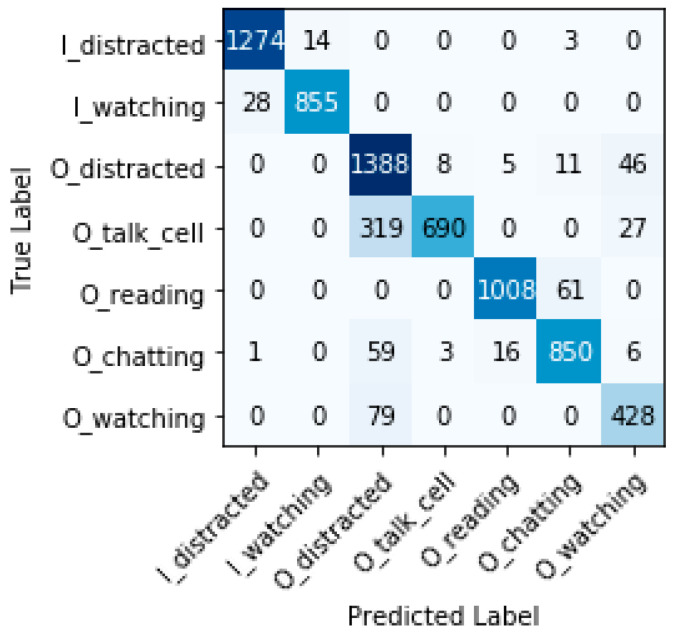
Confusion matrix Inception-v3.

Figure 12, Figure 13 and Figure 14 show the confusion matrices for each model. The main diagonal shows the number of matches found for each category between the true labels (columns) and the predicted labels (rows).

All categories are well predicted. Considering the most relevant categories “In the water watching the children” (I_watching) and “Out of the water watching the children” (O_watching) mentioned above, it is possible to have some wrong predictions, which means that, in some cases, certain distractions have not been detected. The three models sometimes classify distracted behaviors of caregivers as ‘watching the children’ (false positives). These cases represent a risk for children’s safety, but fortunately, do not occur often compared with the true positive values for these categories. Inception-v3 obtains less false positives for I_watching, with 14 versus 27 and 29 cases for VGG-19 and ResNet-50, respectively. ResNet-50 obtains less false positives for O_watching, with 8 versus 21 and 79 cases for VGG-19 and Inception-v3, respectively. We define the false positive rate as subtracting 1 from the specificity or as dividing false positives by the sum of false positives and true negatives. The false-positive rate for I_watching and the three models VGG-19, ResNet-50, and Inception-v3 is 0.43%, 0.46%, and 0.22%, respectively. The false-positive rate for O_watching and the three models (VGG-19, ResNet-50, and Inception-v3) is 0.31%, 0.12%, and 1.18%, respectively. In terms of the false-positive rate, we observe that the obtained values are always very small; VGG-19 and ResNet-50 perform a little worse than Inception-v3 for I_watching. ResNet-50 shows clearly the best results for O_watching.

Furthermore, the three models sometimes classify “watching the children” as distracted behaviors (false negatives). These cases do not pose any risk, but could be annoying for caregivers who are warned to supervise the children when they actually were doing so. ResNet-50 and VGG-19 do not obtain any false negatives for I_watching versus 28 cases for Inception-v3. ResNet-50 and VGG-19 obtain less false negatives for O_watching, with 2 cases each, versus 79 cases for Inception-v3. If we also consider the false-negative rate for the most relevant categories (we define the false-negative rate as subtracting one from recall), we can see that, for I_watching and the two models VGG-19 and ResNet-50, it is 0% and, for Inception-v3, it is 3.17%. The false-negative rate for O_watching and the two models VGG-19 and ResNet-50 is 0.39% and, for Inception-v3, it is 15.58%. The false-negative rates obtained are very small (with the exception of the O_watching category for Inception-v3). These results show that, for VGG-19 and ResNet-50, the child drowning prevention system works correctly with a minimal error rate versus Inception-v3.

## 8. Conclusions and Future Work

In this paper, a novel 5G and beyond child drowning prevention system that detects distracted parents or caregivers and alerts them to focus on active child supervision in swimming pools was developed. For this purpose, we evaluated and implemented three well-known CNN models: ResNet-50, VGG-19, and Inception-v3, to process and classify images. The proposed deep CNN models have revealed that they can be used to automatically detect (based on images) possible distractions of a caregiver who is supervising a child and generate alerts to warn them.

The proposed child drowning prevention system can successfully perform a seven-class classification with very high accuracies of 98% for ResNet-50, 94% for VGG-19, and 90% for Inception-v3. VGG-19 and ResNet-50 achieve the same high performance in the most relevant categories I_watching and O_watching, with accuracies of 100% and 99.61%, respectively. For I_watching, the three models achieve an F1-score of 98%. For O_watching, they reach a F1-score of 98%, 99%, and 84% for VGG-19, ResNet-50, and Inception-V3, respectively. In terms of false-positive rate, the obtained values are always very small; VGG-19 and ResNet-50 perform a little worse than Inception-v3 for I_watching. ResNet-50 shows the best results for O_watching. The false-negative rates obtained are also very small (with exception of the O_watching category for Inception-v3). VGG-19 and ResNet-50 models perform quite well with a minimal false-negative rate versus Inception-v3 for I_watching and O_watching of 0% and 0.39%, respectively. ResNet-50, compared with the other models performs a better classification for most categories. According to the results reached in this research, the proposed system was tested in a swimming pool, but we think it could also be implemented even in swimming lakes or beaches to avoid possible child drowning.

On the other hand, special attention must be paid to security/privacy. Although there is no doubt that distracted parent detection can save lives, associated privacy and security issues need to be analyzed to make our child drowning system socially acceptable. These issues include access rights to data (video images), storage of data, security of data transfer, data analysis rights, and the governing policies. The proposed child drowning prevention system may be vulnerable to a variety of active and passive security attacks (such as eavesdropping) with disastrous consequences (especially if unauthorized parties access underage images). For this reason, security and privacy risks should be minimized by applying existing technical solutions such as encryption, authentication mechanisms, and cryptographic access control during data collection and transmission, encryption message digests, and hashing to assure the integrity of data during data storage and processing. In addition, further works are also required to maintain the security and confidentiality of data by introducing advanced encryption-based techniques. All of these security and privacy challenges must be addressed so that the proposed child drowning prevention system comes out as a promising way to increase swimming pool safety.

We can define the total reaction time as the time elapsing from an observation (image), its transmission to the edge server, the image processing for activity recognition, and the transmission of an alert (if necessary) based on the observation ($D=D_{UE}+D_{Uplink\;}+D_{processing\;}+D_{Downlink\;}).$ As future work, we would like to run the entire system (processing of the images with the neural network and transmission using 5G) in real time. The expected response time for our child drowning prevention system would be around twenty milliseconds (see Table 1). Neural networks have an infinitesimal response time once the weights and the topology have been defined [44]. Further, 5G has been designed to address the requirements of ultra reliable and low-latency communications (URLLC). URLLC has stringent requirements for capabilities such as latency, reliability, and availability. Some use cases include wireless control of industrial manufacturing or production processes, remote medical surgery, and transportation safety. Therefore, 5G is the appropriate technology for our use case.

## Figures and Tables

**Table 1:** Main KPIs for child drowning prevention.

	End-to-End Latency	Data Rate (Uplink/Downlink)	Reliability
5G-enabled child drowning prevention system	20 ms	40 Mbit/s for one video camera/1 Mbps for remote control	99.999%

**Table 2:** Architectures of the three CNN models.

Input	VGG-19 Image	ResNet-50 Image	Inception-v3 Image
Convolutional part	conv3-64 conv3-64 max pooling layer conv3-128 conv3-128 max pooling layer conv3-256 conv3-256 conv3-256 conv3-256 max-pooling layer conv3-512 conv3-512 conv3-512 conv3-512 max-pooling layer conv3-512 conv3-512 conv3-512 conv3-512 max-pooling layer	conv7-64, s = 2 max pooling layer [conv1-64; conv3-64; conv1-256]–[ conv1-64] 2 blocks of [conv1-64; conv3-64; conv1-256] [conv3-128, s = 2; conv1-128; conv1-512]–[conv3-128, s = 2] 3 blocks of [conv1-128; conv3-128; conv1-512] [conv1-256, s = 2; conv3-256 conv1-1024]–[conv1-256, s = 2] 5 blocks of [conv1-256 conv3-256 conv1-1024] [conv1-512, s = 2; conv3-512; conv1-2048]–[conv1-512, s = 2] 2 blocks of [conv1-512 conv3-512 conv1-2048] global_average-pooling layer	Conv3-32, s = 2 Conv3-32 Conv3-64 max pooling layer Conv3-80 Conv3-192, s = 2 max pooling layer Inception A-256 Inception A-288 Inception A-288 Reduction A-768 Inception B-768 Inception B-768 Inception B-768 Inception B-768 Reduction B-1280 Inception C-2048 Inception C-2048 global_average-pooling layer
MLP classifier	FC layer-4096 FC layer-4096 FC layer-07	FC layer-2048 FC layer-2048 FC layer-07	FC layer-2048 FC layer-2048 FC layer-07

**Table 3:** Analysis of both loss and accuracy metrics together.

	Low Loss	High Loss
**Low Accuracy**	A lot of small errors	A lot of big errors
**High Accuracy**	A few small errors	A few big errors

**Table 4:** Accuracy and loss for VGG-19, ResNet-50, and Inception-v3 model.

Models	Training		Testing	
	Accuracy	Loss	Accuracy	Loss
VGG-19	0.9987	0.0056	0.9445	0.0039
ResNet-50	0.9973	0.0110	0.9803	0.3203
Inception-v3	0.9993	0.0019	0.9044	0.0364

**Table 5:** Accuracy of each model with each category.

Parent Status	VGG-19 Accuracy (%)	ResNet-50 Accuracy (%)	Inception-v3 Accuracy (%)	Total Samples
I_distracted	98.99	97.75	98.68	1291
I_watching	100	100	96.83	883
O_distracted	92.32	95.4	95.2	1458
O_talk_cell	98.75	100	66.6	1036
O_reading	100	97.85	94.29	1069
O_chatting	72.73	97.97	90.91	935
O_watching	99.61	99.61	84.42	507

**Table 6:** Evaluation metrics of the VGG-19 model.

Category	Precision	Recall	F1-Score	Total Samples
I_distracted	0.99	0.99	0.99	1291
I_watching	0.98	1.00	0.98	883
O_distracted	0.96	0.92	0.96	1458
O_talk_cell	0.99	0.99	0.99	1036
O_reading	0.87	1.00	0.87	1069
O_chatting	0.84	0.73	0.84	935
O_watching	0.98	1.00	0.98	507

**Table 7:** Evaluation metrics of the ResNet-50 model.

Category	Precision	Recall	F1-Score	Total Samples
I_distracted	1.00	0.98	0.99	1291
I_watching	0.97	1.00	0.98	883
O_distracted	0.99	0.95	0.97	1458
O_talk_cell	0.95	1.00	0.98	1036
O_reading	1.00	0.98	0.99	1069
O_chatting	0.96	0.98	0.97	935
O_watching	0.98	1.00	0.99	507

**Table 8:** Evaluation metrics of the Inception-v3 model.

Category	Precision	Recall	F1-Score	Total Samples
I_distracted	0.98	0.99	0.98	1291
I_watching	0.98	0.97	0.98	883
O_distracted	0.75	0.95	0.84	1458
O_talk_cell	0.98	0.67	0.79	1036
O_reading	0.98	0.94	0.96	1069
O_chatting	0.92	0.91	0.91	935
O_watching	0.84	0.84	0.84	507

## Data Availability

Not applicable.
